# Time from Illness Onset to Death, 1918 Influenza and Pneumococcal Pneumonia

**DOI:** 10.3201/eid1502.081208

**Published:** 2009-02

**Authors:** Keith P. Klugman, Christina Mills Astley, Marc Lipsitch

**Affiliations:** Emory University, Atlanta, Georgia, USA (K.P. Klugman); University of Witwatersrand, Johannesburg, South Africa (K.P. Klugman); Harvard School of Public Health, Boston, Massachusetts, USA (C.M. Astley, M. Lipsitch); Children’s Hospital, Boston (C.M. Astley)

**Keywords:** influenza, pneumococcal, pneumonia, pandemic, 1918, letter

**To the Editor**: Brundage and Shanks (*1*) have studied time to death from the onset of influenza symptoms during the 1918 pandemic in military and civilian populations and found a median time to death of 7–11 days. They argue that these data support the idea that the deaths may be predominantly due to bacterial superinfection after the acute phase of influenza. We observed a similar 10-day median time to death among soldiers dying of influenza in 1918 (*2*), a finding consistent with the time to death for a bacterial superinfection, specifically pneumococcal bacteremic pneumonia (*3*).

The major bacterial pathogen associated with influenza-related pneumonia in 1918 was *Streptococcus pneumoniae* (*1*,*3*). Neither antimicrobial drugs nor serum therapy was available for treatment in 1918. To further analyze the time course of death from influenza in relation to that of pneumococcal pneumonia in 1918, we examined data collected by Tilghman and Finland (*4*) from the pre–antimicrobial drug era of the 1920s and 1930s. The Figure shows the distribution of time from onset of illness to death due to influenza-related pneumonia in 1918 compared with time to death due to untreated pneumococcal pneumonia in the 1920s and 1930s. The Figure indicates a close concordance of the times to death. Similar times to death do not prove the specific bacterial etiology of the 1918 deaths. However, pneumococcal bacteremia was associated with most of the pneumonia deaths reported by Tilghman and Finland (*4*), and most 1918 influenza-related deaths were due to bacterial pneumonia (*5*). Also, up to 50% of patients dying from pneumonia in 1918 had pneumococcal bacteremia (*3*). These similar times to death provide additional evidence that the influenza-related pneumonia deaths during the 1918 influenza pandemic were largely due to the pneumococcus.

**Figure:**
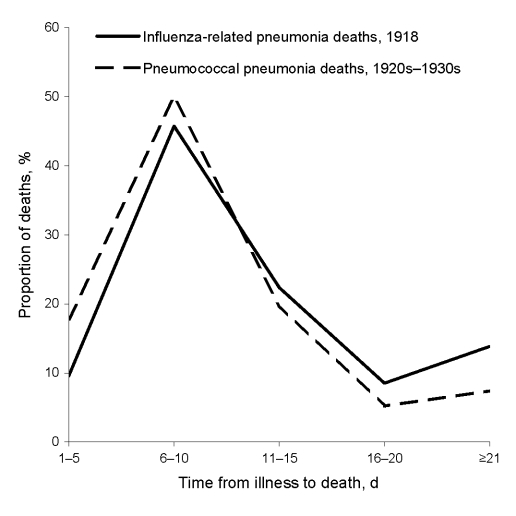
Distribution of days of illness before death from influenza-related pneumonia, 1918, and from untreated pneumococcal pneumonia, 1920s and 1930s.

## References

[1] Brundage JF, Shanks GD. Deaths from bacterial pneumonia during 1918–19 influenza pandemic. Emerg Infect Dis. 2008;14:1193–9. 10.3201/eid1408.071313 18680641 PMC2600384

[2] Mills CE, Robins JM, Lipsitch M. Transmissibility of 1918 pandemic influenza. Nature. 2004;432:904–6. 10.1038/nature03063 15602562 PMC7095078

[3] Klugman KP, Madhi SA. Pneumococcal vaccines and flu preparedness. Science. 2007;316:49–50. 10.1126/science.316.5821.49c 17412937

[4] Tilghman RC, Finland M. Clinical significance of bacteremia in pneumococcal pneumonia. Arch Intern Med. 1937;59:602–19.

[5] Morens DM, Taubenberger JK, Fauci AS. Predominant role of bacterial pneumonia as a cause of death in pandemic influenza: implications for pandemic influenza preparedness. J Infect Dis. 2008;198:962–70. 10.1086/591708 18710327 PMC2599911

